# An evaluation of patient comfort levels during expression with a modified pumping program: a prospective proof of concept study

**DOI:** 10.3389/fgwh.2024.1378263

**Published:** 2024-04-19

**Authors:** T. M. Nicole Manshanden, Danielle K. Prime, Fedde Scheele, Joost Velzel

**Affiliations:** ^1^ Department of Obstetrics & Gynaecology, Northwest Clinics, Alkmaar, Netherlands; ^2^ Research and Development, Medela AG, Baar, Switzerland; ^3^ Department of Obstetrics & Gynaecology, OLVG Hospital, Amsterdam, Netherlands; ^4^ Faculty of Science, Athena Institute, VU University, Amsterdam, Netherlands

**Keywords:** breastfeeding, initiation breastfeeding, breast milk expression, postpartum, breast pump, comfort

## Abstract

**Introduction:**

This study aimed to assess if the implementation of a gentle transition of vacuum mode into a breast pump suction pattern commonly used to initiate milk production would improve user comfort while expressing during the first four days postpartum.

**Methods:**

This prospective study was conducted at OLVG hospital in the Netherlands in two sequential phases. Breastfeeding patients delivering >36 weeks gestation with an infant aged ≤96 h old and a clinical indication to express milk with a breast pump were recruited. Intervention group 1 (*n* = 40) used a hospital-grade electric breast pump with a standard breast pump suction pattern. Intervention group 2 used a hospital-grade electric breast pump with a modified breast pump suction pattern (*n* = 40). The primary outcome was an objective assessment of comfort as measured by participants' need to reduce vacuum level during the 20 min test session. Secondary outcomes included the total expression volume (ml) in 20 min pumping.

**Results:**

The study found that the primary outcome of comfort was significantly improved with the modified breast pump suction pattern compared to the standard pattern (OR 1.29, 95% CI 1.08 to 1.6) with 86% vs. 67% of participants not needing to reduce applied vacuum levels. The amount of milk expressed did not differ significantly between phases (group 1: 7.6 ml (2.7–25.5 ml), group 2: 12.0 ml (1.2–31.5 ml), *p* = 0.43).

**Discussion:**

This study is the first to demonstrate an improvement in user comfort driven by the implementation of gentle transitions in vacuum modes in a commonly used breast pump suction pattern. Research into this novel population combining both pumping and breastfeeding in the first days after birth offers new unique insights on the requirements of breast pump suction patterns.

**Trial registration:**

Registered on clinical trials.gov NCT04619212. Date of registration November 6, 2020.

## Introduction

The World Health Organization (WHO) recommends infants initiate breastfeeding within the first hour after birth and be exclusively breastfed for the first six months of life (1). Medical conditions can lead to parent-infant separation and the need to express breast milk to support the initiation and maintenance of lactation. Parent-infant separation due to infant prematurity is a key reason why most research on breast pump pumping patterns has been conducted in Neonatal Intensive Care Units (NICU) with pump-dependent patients (2–5). There has been limited research conducted with patients in the perinatal ward during the first days postpartum where both breastfeeding and pumping is occurring in combination due to clinical indications such as ineffective infant feeding or infant weight gain issues.

Discomfort and pain during breastfeeding in the first days post-partum are commonly reported (6–8). The incidence of nipple pain peaks typically between the third and seventh day post partum whereafter it improves (9–11). Unlike pump-dependent patients who are unable to feed their infant at breast, breastfeeding patients who are pumping for a clinical indication in the first week post-delivery may have a different experience with a breast pump suction patterns due to nipple skin changes, increases in sensitivity and pain associated with infant sucking in the first week post partum (10). It is known that breast pumping individuals can report the pumping experience as uncomfortable or painful, but it remains unclear how issues related to ineffective or improper infant sucking and increased nipple sensitivity impact the comfort of breast pump suction patterns (12).

Differences in frequency and strength of breast pump suction patterns are perceived by users during pumping, with differing vacuum curve shapes eliciting a range of positive and negative maternal responses (13, 14). Effective milk removal has been demonstrated when the individual's highest comfortable vacuum level is used, however, this maximum chosen level can differ between vacuum curve shapes and frequencies (14, 15). While a pump-dependent population found the pre-programmed changes in rhythm of a commonly used breast pump suction pattern desirable and comfortable, this has not been studied in a mixed breastfeeding and pumping population that may have heightened nipple sensitivity (2).

In daily practice, observations by our clinical staff at our perinatal ward identified potential for improvement for the commonly used breast pump suction pattern when used in this mixed breastfeeding-pumping population. Two moments where the pump suction pattern switched from faster stimulation rhythms to slower expression rhythms were associated with patient discomfort, and the clinical staff observed the need to reduce applied vacuum level. This study aimed to assess if comfort could be improved for mixed breastfeeding and pumping patients who were pumping for a clinical indication by implementing a gentle transition of vacuum level into a breast pump suction pattern commonly used to initiate milk production.

## Materials and methods

### Study setting

This prospective study was conducted at OLVG hospital in the Netherlands between January 2021 and September 2021. The OLVG hospital is an accredited Baby-Friendly Hospital Initiative (BFHI) hospital where infants born after 36 weeks of gestational age stay on the perinatal ward. Home-based maternity care with specialized nurses is standard in The Netherlands, as such uncomplicated deliveries with no medical indication to stay on the perinatal ward are discharged from hospital within six hours postpartum. In the case of infants with mild problems and/or maternal pathology, both patients are admitted to the local perinatal ward until considered safe for discharge it is these patients that make up the recruitment pool for this study.

### Study design and participants

All patients intending to breastfeed are recommended to breastfeed frequently (8 or more times per 24 h). For patients admitted to the perinatal ward, if a clinical indication to start pumping arises, a double-electric pump is used in combination with breast massage. Indications to pump include the need for supplemental feeding (e.g., infant hypoglycemia, infant weight gain issues 7% birthweight drop within 48 h postpartum, or 10% birthweight drop after 48 h postpartum), infrequent feeding (e.g., latching less than eight times within 24 h), maternal indications (e.g., postpartum hemorrhage, nipple trauma, painful breastfeeding or maternal request).

Inclusion criteria included biological women aged ≥18 years, infant aged ≤96 h old with a clinical indication to express milk with a breast pump. Eligible participants intended to provide breastmilk for their infant and in the last 24 h at least 50% of the feedings for the infant were breastfeeds. Exclusion criteria included biological women who were exclusively pumping, had mastitis, received morphine pain medication in the last 8 h, were breastfeeding a previous child or had been breastfeeding in the last 6 months.

This prospective study was conducted in two sequential phases, intervention 1 followed by intervention 2. When the planned sample size per day postpartum were reached for intervention group 1, recruitment for intervention group 2 began. Unique participants were recruited for an assessed breast pumping session when the clinical team flagged their need for a clinically indicated pumping session. For the test session, intervention group 1 used a hospital-grade electric breast pump with a standard breast pump suction pattern. Intervention group 2 used a hospital-grade electric breast pump with a modified breast pump suction pattern.

### Intervention

Intervention group 1 expressed using the Symphony breast pump with the INITIATE and MAINTAIN programs currently available (Medela AG, Baar, Switzerland). Intervention group 2 expressed using the Symphony breast pump with a modified INITIATE program (INITIATE 2.0) and standard MAINTAIN program. The INITIATE 2.0 program (Medela AG, Baar, Switzerland) was modified to include a gentle transition in vacuum ramp-up over approximately 6 vacuum cycles at two timepoints where a pre-defined change in suction rhythm occurs from stimulation to expression patterns. At the beginning of the expression session, the researcher helped the patient adjust vacuum to their maximum comfortable vacuum setting. During the pumping session manual adjustment of this vacuum level was possible. A data logger (Medela AG, Baar, Switzerland) was attached to the breast pump to objectively record the vacuum levels used throughout the session and identify any manual adjustment of vacuum. Expressed milk volume was assessed by weighing the pump set before and after the expression session. Each expression session ran for a total of 20 min, 15 min with the INITIATE (standard or modified) and 5 min with MAINTAIN.

### Outcomes

For each participant, the following participant characteristics were recorded: postpartum inclusion day, mode of delivery, parity, multifetal pregnancy, prior experience breastfeeding, prior experience pumping, indication to pump, breast and nipple situation since delivery and time since last milk removal. After an expression session, participants filled in a questionnaire on level of comfort and experience. Clinical staff filled in a questionnaire on their observations.

The primary outcome was an objective assessment of comfort measured as participants not needing to reduce vacuum level during either of the two adjusted vacuum transitions and was measured via the data logger. Secondary outcomes included total expression volume in milliliters as well as qualitative assessment of comfort and satisfaction by questionnaire for participant (comfort and satisfaction). After the test session clinical staff answered the question “Did the mom experience any pain or discomfort during pumping, particularly during the change in the pumping pattern sections?” (No, Yes—If yes score 1 -mild to 7- severe).

### Statistical analysis

A sample size calculation was performed for the objective measure of comfort while pumping as measured by the need to change vacuum at the two identified transitions in the breast pump suction pattern. Available data from clinical staff observations suggested that 44% (7 out of 16) of mixed breastfeeding-pumping patients needed to change pressure when using the current INITIATE program. Due to the changes to the program, it was assumed that no patient would need to change the vacuum, however, a conservative value of 10% was used for the sample size calculation. Samples were drawn from a binomial distribution according to those proportions and compared with a *χ*^2^ test, using an alpha of 0.05. Power was estimated based on 1,000 simulations. A sample size of 32 participants per group (64 in total) yielded a power of at least 80%. Furthermore, we intended to recruit a balanced sample by days postpartum as nipple sensitivity is reportedly more frequent from day three postpartum (10). Two additional participants were allocated per day post-partum to account for distribution errors and withdrawals resulting in 40 participants per group. Specifically, *n* = 5, *n* = 10, *n* = 15 and *n* = 10 participants on days 1, 2, 3 and 4 post-partum, respectively, per group.

Binary outcomes were tested with *χ*^2^ tests and fisher exact tests. The outcomes were presented with counts and percentages in each category. Continuous variables were compared with *t*-tests or non-parametric tests as appropriate. Means and standard deviations, or medians and 25th and 75th percentiles were presented as appropriate to the test used, together with the relevant difference.

Secondary analyses to assess the robustness of our findings were performed in which the need for manual adjustment of vacuum analyses were adjusted for any potential confounding variables. In order to account for the repeated measures nature of the overall comfort endpoint a Generalised Estimating Equation with a binomial link (GEE) test was used.

### Ethics and role of sponsor

Prior the start of the study, a local ethics committee approved the study protocol and the study was registered on clinical trials.gov NCT04619212. The trial was performed according to the principles of ICH-good clinical practice. Before any study specific procedure was performed informed consent was obtained. This study was conducted with the support of Medela. It was explicitly agreed that no financial funding was to be received by the study site.

## Results

A total of 80 participants were recruited in two sequential phases, intervention group 1 (*n* = 40) and intervention group 2 (*n* = 40). For one subject in intervention group 1 the incorrect pumping program was chosen. Therefore this subject was excluded from all analysis. No other participants required exclusion from the data analysis, and no adverse events occurred.

Baseline characteristics are shown in Table 1, there were no significant differences between intervention groups for mode of delivery, parity, multifetal pregnancy and prior experience of breastfeeding and pumping. There were no significant differences between interventions for the indication to use a breast pump. The most frequent indication was formula supplementation (25 participants in group 1 and 34 participants group 2). Maternal indications included one maternal request to pump in group 2, and 3 participants in group 1 for pain. Twelve participants in intervention group 1 experienced engorgement of the breast since delivery compared to four participants in group 2 (respectively 31% vs. 10%, *p* = 0.04). Fewer participants experienced no breast/nipple situations since delivery in group 1 (respectively 64% vs. 88%, *p* = 0.02). The time (minutes) since last milk removal was similar (*p* = 0.44) between group 1 [60 (IQR: 34–133), *n* = 39] and group 2 [46 (IQR: 31–128), *n* = 40].

**Table 1:** Participant characteristics.

	Total (*N* = 79)	Intervention group 1 (*N* = 39)	Intervention group 2 (*N* = 40)	*p* value
Vaginal delivery	44 (56%)	22 (56%)	22 (55%)	0.92
Primiparous	60 (75%)	29 (74%)	31 (78%)	0.95
Multifetal pregnancy	3 (4%)	1 (3%)	2 (5%)	0.98
No prior experience breastfeeding	62 (78%)	31 (79%)	31 (78%)	0.95
No prior experience pumping	61 (77%)	30 (77%)	31 (78%)	0.84
Postpartum day of inclusion				
Day 1	9 (11%)	4 (10%)	5 (13%)	
Day 2	21 (26%)	10 (26%)	11 (28%)	
Day 3	29 (36%)	15 (38%)	14 (35%)	
Day 4	20 (25%)	10 (26%)	10 (25%)	
Indication to pump				
Neonatal indication	74 (94%)	35 (90%)	39 (98%)	0.34
– Infant formula supplementation	59 (75%)	25 (64%)	34 (85%)	0.06
– Infant feeding is ineffective	48 (60%)	21 (54%)	27 (68%)	0.31
– Infant weight gain issues	5 (6%)	4 (10%)	1 (3%)	0.34
Maternal indication	4 (5%)	3 (8%)	1 (3%)	0.60
Breast situation since delivery				
Nipple soreness	42 (53%)	21 (54%)	21 (53%)	0.91
Engorgement	16 (20%)	12 (31%)	4 (10%)	0.04
Nipple trauma	5 (6%)	5 (13%)	0 (0%)	0.03

**Figure 1:**
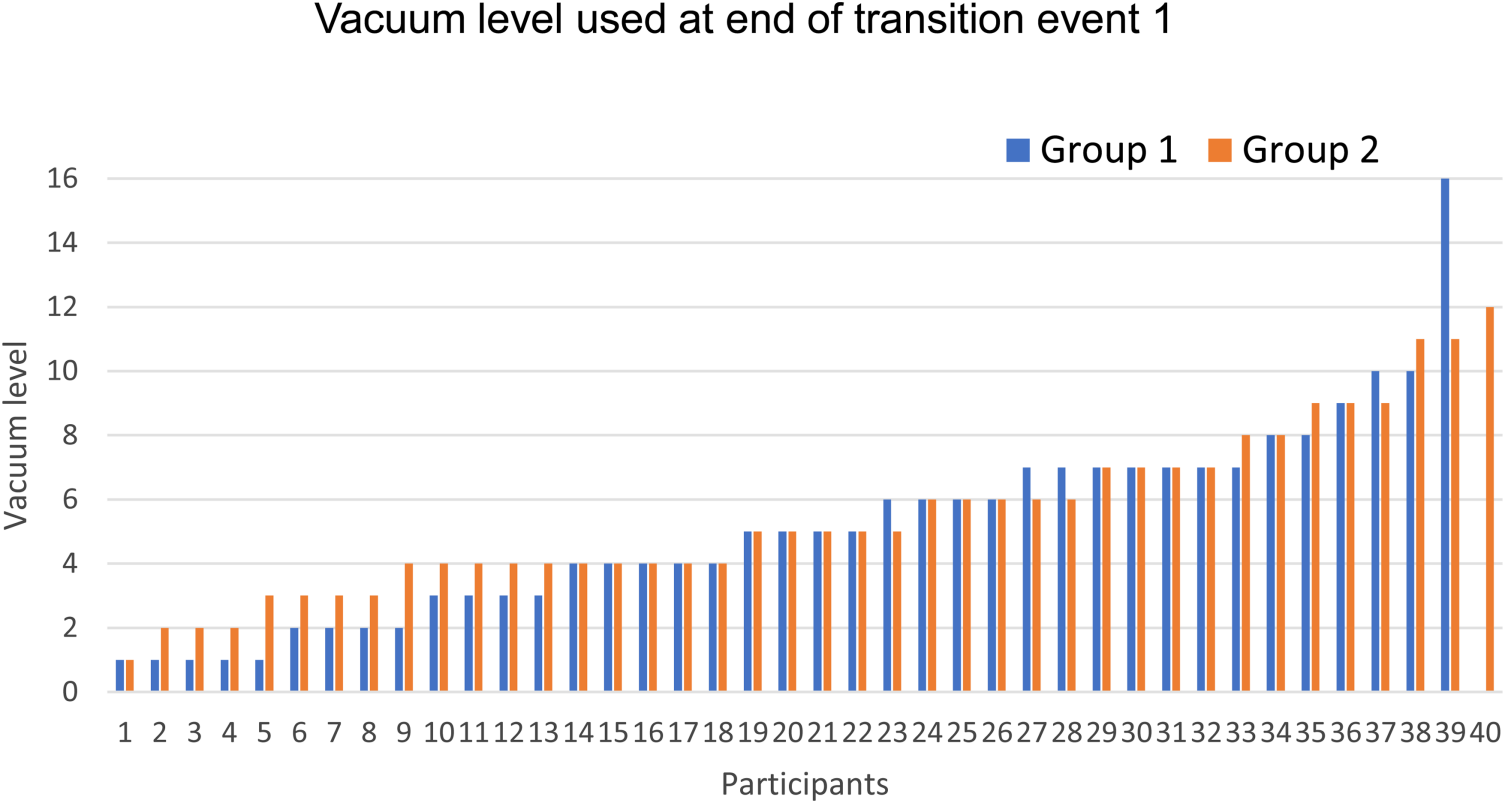
Distribution of vacuum levels chosen at the end of the transition event 1 for intervention group 1 (blue, *n* = 39) and intervention group 2 (orange, *n* = 40). Events are ordered from lowest to highest recorded vacuum level.

Table 2 presents the primary and secondary outcomes. In group 1, 67% of the participants did not need to reduce applied vacuum level, whereas in group 2, 86% of the participants did not need to reduce applied vacuum level at the transitions (OR 1.29, 95% CI 1.08 to 1.55, *p* = 0.01). The amount of expressed milk did not differ significantly between intervention groups (7.6 ml for group 1 and 12.0 ml for group 2, *p* = 0.43). The distribution of vacuum levels chosen at the end of transition event 1 is described in Figure 1. Overall, nearly half of the participants used level 4 or less (group 1 *n* = 18 (46%), group 2 *n* = 18 (45%)), equivalent to a vacuum ranging from −90 to −130 mmHg. For those using level 4 or less, there was a significant (*p* = 0.04) increase in the average level chosen from group 1 (2.5 ± 1.2) to group 2 (3.3 ± 1.0).

**Table 2:** Primary and secondary outcomes.

Manual adjustment of vacuum level	Intervention group 1 (*N* = 39)	Intervention group 2 (*N* = 40)	Relative Risk (95% CI)	*p* Value
Participants not reducing vacuum level in whole pumping session (%)	52 (67%)	69 (86%)	1.29 (1.08, 1.55)	0.013
– Participants not reducing vacuum level in event 1	24 (62%)	32 (80%)	1.31 (0.98, 1.75)	0.055
– Participants not reducing vacuum level in event 2	28 (72%)	37 (93%)	1.29 (1.04, 1.60)	0.014
Median expression volume in ml (IQR)	7.6 ml (2.7–25.5 ml)	12.0 ml (1.2–31.5 ml)	NA	0.43

To confirm the robustness of the findings, secondary sensitivity analyses were performed (Table 3). Interventions were compared for the primary outcome (any manual adjustment of vacuum) using generalised estimating equations to account for the repeated measures nature of the data. Analyses were adjusted for the potential confounding variables indication to pump, and breast situation. Breast situation was summarised by engorgement (yes/no) and no issue (yes/no). Since trauma was rare, and to avoid multicollinearity it was not included. After adjustment for indication to pump, the odds ratio for not reducing vacuum level was OR 1.20 (95% CI 1.02 to 1.42, *p* = 0.03). After adjustment for indication to pump and breast situation since delivery, the odds ratio for not reducing starting vacuum level was OR 1.19 (95% CI 1.01 to 1.40, *p* = 0.04).

**Table 3:** Secondary sensitivity analyses for objective outcomes.

Manual adjustment of vacuum level	Intervention group 1 (*N* = 39)	Intervention group 2 (*N* = 40)	aOR^a^ (95% CI)	*p* Value	aOR^b^ (95% CI)	*p* Value
Participants not reducing vacuum level in whole pumping session (%)	52 (67%)	69 (86%)	1.20 (1.02, 1.42)	0.03	1.19 (1.01, 1.40)	0.04
– Participants not reducing vacuum level in event 1	24 (62%)	32 (80%%)	1.20 (0.98, 1.45)	0.08	1.22 (1.00, 1.49)	0.05
– Participants not reducing vacuum level in event 2	28 (72%)	37 (93%)	1.21 (1.02, 1.44)	0.03	1.16 (0.98, 1.38)	0.10

^a^ Adjusted for indication to pump.

^b^ Adjusted for indication to pump and breast situation since delivery.

**Figure 2:**
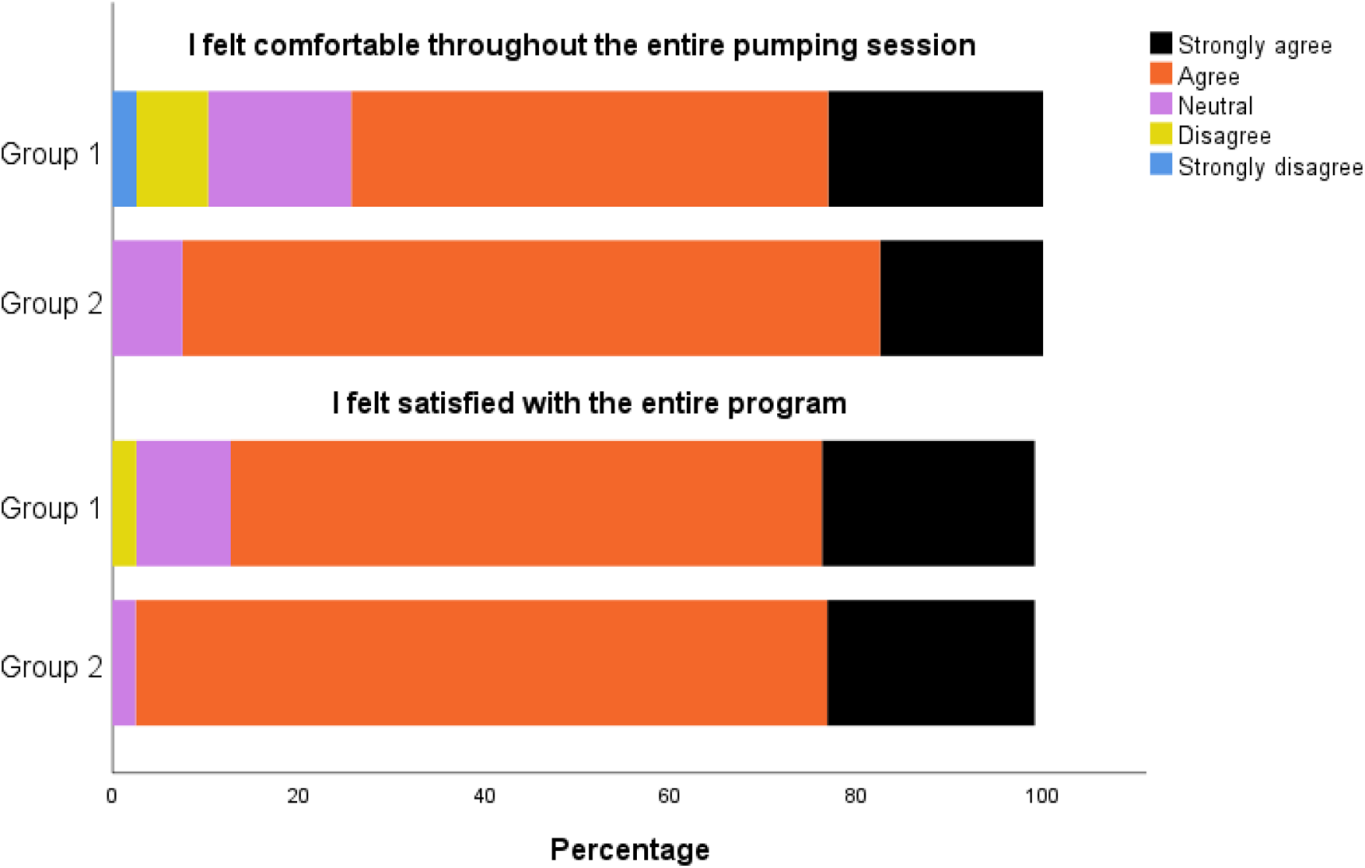
Post-session participant feedback for group 1 (*n* = 39) and group 2 (*n* = 40) regarding comfort and satisfaction with the pumping program.

Likert scales on participant feedback are demonstrated in Figure 2. The majority of the participants in both groups agreed or strongly agreed on feeling comfortable during pumping (respectively 74% vs. 93%, *p* = 0.42). Clinical staff observational feedback for group 1 showed that 17 (44%) participants experienced no pain or discomfort during pumping and 35 (88%) participants for group 2 (*p* < 0.01).

## Discussion

This mechanistic, pre-market proof of concept study shows that the modified breast pump suction pattern was more comfortable and convenient for use with mixed breastfeeding-pumping persons in the first days post-delivery without an apparent compromise to milk output when a clinical indication to pump arose.

To the best of our knowledge, studies aiming to optimise acceptability and comfort of breast pumping programs for our study population have not yet been undertaken. Our in-patient population delivered their infants at term, intended to and initiated breastfeeding, and subsequently needed to use a breast pump in addition to breastfeeding due to one or more clinical indications according to our local protocol. It is important to note that the majority of uncomplicated deliveries in our facility are discharged within six hours postpartum, our study population therefore represents complicated deliveries and/or patients with medical indications to remain admitted on the perinatal ward. The time of use of the breast pump was in the first days post-partum, specifically during the critical days of more nipple sensitivity, prior to secretory activation or milk “coming in”, when the role of the pump is to support the initiation of lactation and removal of colostrum.

This study has the limitations that it was not randomized or blinded, however, a significant improvement in comfort was found even after secondary sensitivity analyses for robustness. These findings were achieved through a combination of objective data logger and subjective feedback from attending Lactation Consultants. The primary outcome was collected objectively via a data logger which tracked all events where vacuum was adjusted. This ensured that the results did not rely on subjective feedback of the participants or staff alone. The subjective feedback that was collected from attending clinical staff matched the primary outcome well with significantly more events of pain or discomfort described for intervention group 1 compared to intervention group 2.

The majority of current literature regarding comfort and effectiveness of pumping has been conducted within pump-dependent populations (2, 16–18). Research into this novel population combining both pumping and breastfeeding offers new unique insights on the requirements of breast pump suction patterns. Infant sucking at the breast in the first week post-partum has been associated with nipple skin changes, increases in sensitivity and pain (10). Literature suggests that the implications of breastfeeding initiation problems could impact breast pumping experiences remain unclear. However, it is feasible that sore nipples, resulting from ineffective or improper infant sucking, could be exacerbated by pump use, potentially leading to increased reports of nipple soreness as a pump-related problem (12). In our study, 60% reported ineffective infant feeding, and 53% reported nipple soreness. This increased nipple sensitivity is likely to directly impact the acceptability of breast pump suction patterns, and in particular the rating of comfort, compared to pump-dependent populations. Our findings support this, whereby modification of the pumping pattern by implementation of gentle transitions between the rhythm changes resulted in a significant reduction in the need to reduce vacuum and thereby a reduction in discomfort felt by the patients. Further to this, a strength of our approach is that we excluded participants which received morphine pain medication in the last 8 h to ensure that any discomfort experienced during pumping was not masked by pain medication.

In addition, this mechanistic study provided crucial understandings into vacuum levels (mmHg) in breast pumps during the different lactation stages. Established lactation users typically select −190 mmHg, while early lactation pump users have been less well characterised (15, 19). One study implemented a pumping intervention for breastfeeding patients 24 h after caesarean section with vacuum of −100 mmHg. While the study used an older pump technology and pump suction pattern, patients reported a higher breast pain score in the pumping intervention group, suggesting discomfort even with weak vacuums (20). Another study identified that infants born after caesarean section applied lower vacuums (−103.35 mmHg) compared to vaginally delivered infants (−149.18 mmHg), and that the lower suction level may be associated with delayed secretory activation (21). That same group then applied a pumping intervention with either −100 mmHg or −150 mmHg, those receiving −150 mmHg were found to have a faster onset of secretory activation (22). As there may be an association between lactation outcomes and targeting vacuum levels towards −150 mmHg, offering suction pattern designs that support maintaining the set vacuum level is positive in this direction. In our study, nearly half of the patients utilised vacuum levels 1 to 4, and these users maintained higher vacuums with the modified program indicating that the modification is particularly beneficial for sensitive users expressing in the −90 to −130 mmHg vacuum range.

While participants in this study were still in the colostrum phase, and this study is not adequately powered to assess differences in milk output it is reassuring to note that the exploratory outcome milk expression volumes were not obviously different between groups. As this study has a mechanistic design, future studies should focus on milk output and other lactation outcomes with the modified program considered clinical relevance and clear description of its population.

The vast positive short- and long-term health infant and parent are well known (23). In high impact literature, multiple series have confirmed these benefits, including global economic savings (23–27). Yet there remains an overwhelming influence of commercial milk formula marketing and rates of breastfeeding globally vary wildly. In low-income countries, most infants are still breastfed at 1 year, compared with less than 20% in many high-income countries and less than 1% in the UK (23). Therefore, research efforts advocating for improving breastfeeding and pumping experiences need to be widely encouraged to help with achieving the target of exclusive breastfeeding for six months. Future studies need to strive for standardised metrics and protocols, establishing global definitions and a call for development of a core outcome set. This study is grounded in clinical observations from a teaching hospital in Amsterdam ensuring its clinical relevance and applicability to a wide range of healthcare settings, offering a foundation for generating further evidence in this crucial area of parent and infant health.

## Conclusion

Modification of the INITIATE program with the implementation of gentle transitions between when the pattern switches from simulation rhythms to expression rhythms has been shown to increase comfort during pumping for study participants.

## Data Availability

The raw data supporting the conclusions of this article will be made available by the authors, without undue reservation.
